# European seabass, *Dicentrarchus labrax*, show no significant response to infrared light

**DOI:** 10.1111/jfb.70038

**Published:** 2025-04-01

**Authors:** Alexa Sugpatan Abangan, Victor Simon, Fabien Morandeau, François Martignac, Dorothée Kopp, Robin Faillettaz

**Affiliations:** ^1^ DECOD L'Institut Agro, IFREMER, INRAE Lorient France; ^2^ IFREMER University of Brest, CNRS, IRD, LEMAR Plouzané France; ^3^ DECOD L'Institut Agro, IFREMER, INRAE Rennes France

**Keywords:** animal trajectory, fish behaviour, optomotor response, photokinesis, phototaxis

## Abstract

Infrared (IR) light is widely accepted as a non‐intrusive lighting for discreet observation but relevant studies are scarce. The response of European seabass *Dicentrarchus labrax* (Linnaeus 1758) towards IR light was tested during laboratory experiments alternating sequences of IR light and dark conditions. Swimming trajectories were extracted from hydroacoustic videos and behavioural metrics (e.g. speed, bearings) were quantified from the *D. labrax* movement patterns. *D. labrax* showed no preference nor deterrence towards IR light, supporting the use of discreet IR light as an alternative to visible light for monitoring unbiased *D. labrax* behaviour.

Fish vision is adapted to the light conditions of their natural habitats, which predominantly involves visible light (Lythgoe, 1988). Fish's photoreceptor cells can absorb wavelengths from approximately 350–635 nm (Carleton et al., 2020; Douglas, 2001). Infrared (IR) light, which has wavelengths longer than 700 nm, is beyond the sensitivity range of most fish and does not provide sufficient energy to trigger the necessary chemical changes in the photopigments of fish (Levine & MacNichol, 1982). IR light is therefore commonly used as an alternative to visible light to study fish behaviour in captivity or in situ without bias, to determine responses to ambient or artificial lighting (Pulgar et al., 2019; Ryer et al., 2009; Widder et al., 2005; Yochum et al., 2024) or how fish interact with fishing gears to improve the catch or escapement of individuals (Chladek et al., 2021).

IR vision is an adaptable evolutionary trait found in species inhabiting turbid environments (Matsumoto & Kawamura, 2005; Shcherbakov et al., 2013). Some fish species have been observed to enhance their IR vision for navigation in murkier waters (Matsumoto & Kawamura, 2005), a process facilitated by the enzyme Cyp27c1 linked to vitamin A adaptation (Enright et al., 2015). It therefore appears relevant to confirm the absence of IR response prior to using it as control for fish behavioural responses to different wavelengths.

European seabass, *Dicentrarchus labrax* (Linnaeus 1758), has been the focus of numerous physiological and behavioural studies (Georgopoulou et al., 2021; Karakatsouli et al., 2012; Marchesan et al., 2005; Villamizar et al., 2011). Brill et al. (2019) measured the retinal function of the species and showed that its spectral sensitivity ranges from 300 nm to at least 700 nm but longer wavelengths were not considered.

*D. labrax* is the second most expensive commercial species targeted by several European fleets (EUMOFA, 2021), mostly using handlines hooks, gillnets and bottom trawling (ICES, 2024). They are caught alongside other species (e.g. haddock *Melanogrammus aeglefinus* (L. 1758), whiting *Merlangius merlangus* (L. 1758), common sole *Solea solea* (L. 1758) and Atlantic mackerel *Scomber scombrus* (L. 1758). Hence, the fishing allowance for *D. labrax* also impacts other species. *D. labrax* stocks are expected to pursue their decrease, prompting the need for remedial measures (ICES, 2024). Light is one avenue to selectively fish *D. labrax*, but requires unbiased sampling of behaviour towards light stimuli.

We analysed the movement of *D. labrax* from videos to quantify their behaviour and determine their stimulus response through generic pattern recognition and signal processing techniques. Given that seabass spectral sensitivity for IR light is unknown, we hypothesized that *D. labrax* does not respond to IR light, as in most species (Levine & MacNichol, 1982). While studies have used IR light to monitor *D. labrax* behaviour in captivity (e.g. Benhaïm et al., 2012, 2013; Zhou et al., 2017) the species' response to IR light has never been explicitly explored.

This experiment was preliminary to a larger experiment focused on evaluating whether light stimuli could trigger behavioural responses of *D. labrax* in a fishing context, aiming to improve fishing gear selectivity. The present study was also conducted in accordance with the Protection of Animals used for Scientific Purposes (Article R.214‐120, Decree No. 2013‐118). The protocol was approved by the French Ethics Committee for Animal Experimentation with the reference number 38910_2022101818033034. Every effort was made to minimize stress to the experimental subjects, which is critical both ethically and for the validity of the behaviour observed. The number of individuals exposed to this experimental procedure was limited, in line with the “Three Rs” rule: Replacement, Reduction and Refinement (Directive 2010/63/EU).

A cohort of 15 reared adult *D. labrax* (>4 years old, size range 35–44.2 cm, mean total size 40.46 cm, weight 0.5–1.5 kg, mean weight 1.005 kg), which are expected to have the most developed visual system compared to juveniles (Carleton et al., 2020), was studied to confirm if IR lighting can be used to baseline the species' behaviour prior and after activating a “visible” light stimulus (i.e. *λ* in the range 400–700 nm). The experimental basin was supplied with recirculated seawater with water temperature, pH and oxygenation maintained both at optimal levels (temperature range 12–15°C, pH range 7.6–8, oxygen level >85%). The fish were fed daily with a commercial diet (B‐Repro Marin) and fasted for 24 h pre‐experiment. Each *D. labrax* underwent the experiments individually in a 200 × 200 × 60‐cm basin placed in an area void with light and noise to exclude other sensory cues (Data S1a,b). A hydroacoustic camera (Oculus®, Blueprint Subsea; 1 fps frame rate with a 1.2 MHz frequency) was submerged on one corner, protected by a plastic barrier, with its field of view covering the entire basin area, while maintaining the symmetry of the basin relative to the camera (Data S1c,d). Hydroacoustic cameras utilize undetectable, high‐frequency acoustic signals which capture fish movement without interfering with any other cues (Martignac et al., 2015; Munnelly et al., 2023). Two IR lamps were used as the IR sources (BERSUB® IR Wide, *λ* = 750–900 nm, peak *λ* at near‐infrared = 850 nm; Data S2a,b), located on each side of the basin (Data S1e).

Each *D. labrax* was transferred with a fish net from its rearing basin to the experimental basin (2 m distance; Data S1a,f,g) and acclimatized for 2 min prior to the trial. The rapid transfer without anaesthesia and the short acclimation phase aimed at maintaining a relatively high level of stress, in line with the fishing context, where tested stimuli would be deployed in situ. Each trial then lasted 12 min, split into four 3‐min testing phases. The testing sequences started with a dark phase (OFF^1^), followed by a first IR phase (IR^1^_left_), with IR light on the left side of the basin relative to the hydroacoustic camera), a second dark phase (OFF^2^), then a second IR phase (IR^2^_right_), with IR light on the right side of the basin). Each of the *D. labrax* underwent the experiments only once, with each one transferred to a different rearing basin afterwards to prevent testing the same individual.

Fish trajectories were reconstructed using the fish position per second (Figure 1a). The metrics considered were swimming speed and position. The positional data were then used (1) to determine if each individual displayed a preferred direction during the phases (defined as *directionality [r]*, based on the Rayleigh's *r* statistic computed using each position of the fish during the trial and its mean bearing) and (2) to detect *orientation*, whether all directional individuals (i.e. Rayleigh's *r* test on individual trajectory with p < 0.05) responded similarly to the IR stimuli (defined as *precision [R]*, based on Rayleigh's *R* statistic computed using each mean bearings of all individuals fish during the trial and the group's mean bearing; Batschelet, 1981). Standard circular statistics (Rayleigh's *r*, Watson's *U* ^2^ and Wallraff tests; Batschelet, 1981) were then computed to compare bearings distribution among phases, dark and IR phases, and relative to the basin and IR source.

**FIGURE 1:**
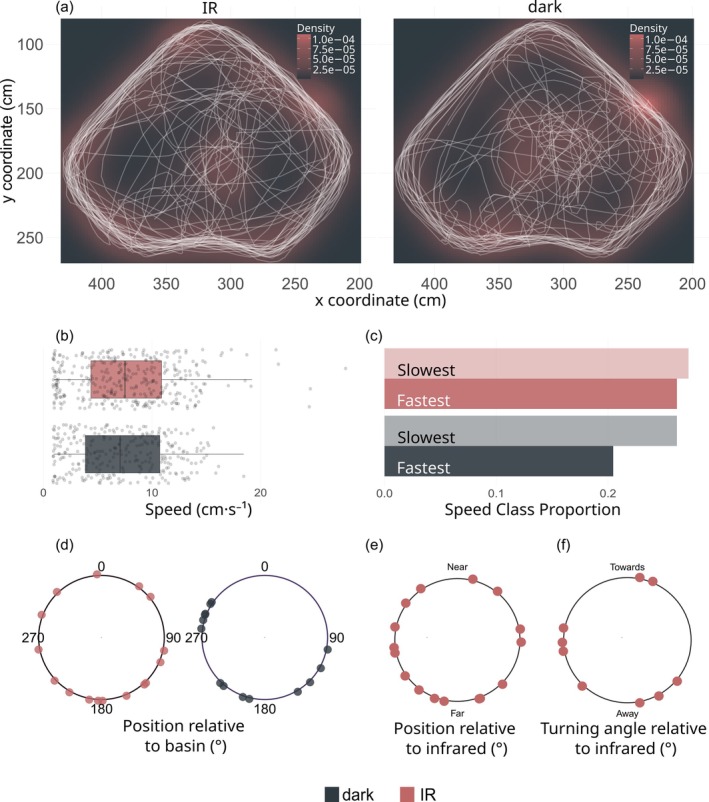
The response profiles of *Dicentrarchus labrax* during the experiment between IR^1‐2^ and dark phases: (a) individual trajectories, (b) speed distribution and (c) proportion of fastest (maximum speed) and slowest (minimum speed) class. Mean bearings of significantly directional individuals only of the (d) positions relative to the basin per phase, IR vs. dark, (e) positions relative to the IR source per phase, IR^1^ and IR^2^, and (f) mean bearings of the swimming directions relative to the IR source per phase, IR^1^ and IR^2^.

To test for any changes in distributions of speed and directionality among the OFF^1^, IR^1^ OFF^2^ and IR^2^ phases, Kruskal–Wallis and Dunn tests with Holm correction were used while Wilcoxon rank sum tests were used to test between IR phases (IR^1‐2^) and dark phases combined. The Wilcoxon rank sum test was again used to test if there was a difference in directionality between the IR^1‐2^ and dark phases. Watson's U test was used to evaluate orientation towards the IR light. Lastly, the Wallraff test (Batschelet, 1981) was used to compare the mean bearings relative to the basin between OFF^1^ and IR^1^, OFF^2^ and IR^2^, IR^1^ and IR^2^, OFF^1^ and OFF^2^, and IR^1‐2^ and dark, as well as between IR^1^ and IR^2^ relative to the IR sources only. The statistical tests for circular data were performed for both the positional angles and swimming directions. As we dealt with a small cohort due to logistical and ethical constraints, we relayed the statistical power of our tests to consider the influence of the sample size (Jennions & Møller, 2003). The data were processed in *R* statistical software (v4.4.0, R Core Team, 202) with the packages *circular v0.5.0* (Lund & Agostinelli, 2004), *discr v0.5* (Irisson, 2015), *dplyr v1.1.4* (Wickham et al., 2023) and *ggplot2 v3.5.1*(Wickham, 2016). Additional information on the study's method is provided in Data S2.

Overall, 10 fishes (66%) displayed swimming patterns, with five individuals that did not move notably during the experiment (Data S3) and were therefore not included in further analyses since circular statistics would not enable us to draw conclusions. For swimming speed, all tests showed no difference between the dark and IR phases (Kruskal–Wallis: $\chi^{2}$ = 0.165, p = 0.685; Figure 1b,c). At the individual level, three fishes showed significant differences in speed distributions between the IR phases (IR^1^ and IR^2^) and between the dark phases (OFF^1^ and OFF^2^; Kruskal–Wallis tests, all three p < 0.05). At the group level, speed distributions did not differ between phases (Kruskal–Wallis test, p = 0.63, power = 0.25; Figure 1b), nor did the proportion of the fastest speed (>9 cm/s) and slowest speed (<4 cm/s) between IR^1‐2^ and dark phases (chi‐squared test, $\chi^{2}$ = 2.67, p = 0.10, power = 0.41; Figure 1c).

For position, all tests also showed random orientation (Watson and Wallraff tests, p > 0.05; Figure 1d–f). At the individual level, nine fishes showed significant directionality in swimming speeds (Rayleigh's test, all p < 0.05). At the group level, the mean positional bearings of the nine individuals that showed significant directionality showed a uniform distribution and were not significantly different from a von Mises distribution when looking at both their directionality in the basin and relative to the IR sources (Watson's *U* ^2^ test, all $U^{2}$∈[0.019,0.153], all p > 0.05). All results did not show any particular distribution that would indicate that there is a preferred direction in the dark or in the IR^1‐2^ phase. The mean positional bearings between all pairs of phases showed no difference in orientation (Wallraff tests between OFF^1^/OFF^2^, IR^1^/IR^2^, OFF^1^/IR^1^, etc.; all $\chi^{2}$∈[0.037,1.103], all p > 0.05). The mean cardinal position showed no preferred direction during the IR^1‐2^ and dark phases (Rayleigh's test, R = 0.461, 0.450 respectively, both p ≥ 0.10) (Figure 1d). The position relative to the IR source both did not show any preferred direction (Rayleigh's test, R = 0.210, 0.482 respectively, both p ≥ 0.178; Figure 1e).

For swimming direction, all tests showed no particular pattern where *D. labrax* swam away from or towards the IR sources (Figure 1f). The results of the statistical tests do not relay any other significant information as compared to the fish's position, with only five individuals displaying directionalities in their swimming direction relative to the basin during at least one phase (Rayleigh's test, p < 0.05). Similar to the positional angles, there was no preferred direction in the dark nor in the IR^1‐2^ phase, regardless of the reference (basin vs. IR; Watson's $U^{2}$ tests, all $U^{2}$ ∈[0.047,0.049], all p > 0.05), nor between phases (Wallraff test, all $\chi^{2}$∈[0.24,2.143], all p > 0.05).

In commercial fishing, the immediate response to light, such as an instantaneous change in speed or direction, or general attraction can give insights on how illumination can be installed in gears as bycatch reduction lights (BRLs) (e.g. Cuende et al., 2022; Lomeli & Wakefield, 2019; Sardo et al., 2020; Southworth et al., 2020; Underwood et al., 2021; Yochum et al., 2022). Nevertheless, BRLs have not yet been applied to seabass fishery, although seabass populations are vulnerable to fishing (Regulation [EU] 2019/1241). Future applications of BRLs may be of interest to seabass fisheries to avoid population decline (Council Regulation [EU] 2024/257). The study of light responses of *D. labrax* in situ and ex situ could require discreet lighting such as IR light, particularly on optical observations.

Overall, our findings indicate that *D. labrax* may not exhibit sustained or significant alterations in behaviour due to IR light alone, and that the IR lights can be used without biasing the *D. labrax* response. Although commonly used, our findings validate the efficiency of IR lights for discreet camera observations of *D. labrax* behaviour in varied lighting conditions, particularly in designing more effective and less intrusive observation and tracking methods in marine research. IR lighting can thus be used to monitor fine‐scale *D. labrax* responses (<2 m), to light stimuli in situ, particularly in trawls (e.g. Williams et al., 2013), and to develop efficient bycatch reduction devices based on genuine fish behaviour (e.g. Olla et al., 2000).

Most studies have used acoustic imaging for testing fish behaviour in dark versus lit conditions (e.g. O'Connell et al., 2014; Rakowitz et al., 2012; Williams et al., 2013). Acoustic imaging was particularly relevant in our study since all individuals were identified, which otherwise limits the study of species‐specific responses (e.g. Becker et al., 2013). Our results therefore comfort the efficiency of acoustic imaging method to assess the effect of light sources on fish behaviour during dark (control) phases.

When concluding on a behavioural response, potential sources of noise and bias must be acknowledged. Statistical testing on a small cohort should be done with caution (Jennions & Møller, 2003; Landler et al., 2018), yet despite the sample size constraints, these exploratory trials all concur and provide a basis to be compared with any succeeding studies. Here, all trials were successful and thus working with 15 individuals was sufficient. Still, in similar experiments, a larger number of individuals would increase the chance of success, in particular if some trials are to be discarded (i.e. stressed individuals, outside nuisances, etc.).

Studying *D. labrax* in captivity should not pose any bias in the inherent capability of detecting light, since the fundamental genetic makeup of a fish's visual systems is preserved across generations, even in captivity (Chittenden et al., 2010). However, when testing with wild *D. labrax*, it is noteworthy that they may show more agitation or relatively faster movements when tested in a controlled environment as opposed to domesticated fish, who may show a reduced startle response (Benhaïm et al., 2012). Moreover, given that changes in fish spectrum sensitivity are only observed during long‐term exposure (i.e. days to weeks) to different light conditions and intensities (Noureldin et al., 2021; Shin & Choi, 2014; Volpato & Barreto, 2001), it is important to acknowledge the potential for fish to adapt to changing environmental conditions, such as habitat shifts or diet changes, which could alter their visual capabilities (Brill et al., 2019).

## AUTHOR CONTRIBUTIONS

All authors conceptualized and designed of the study and built the experimental set‐up. Alexa Abangan, Victor Simon, Fabien Morandeau, Dorothée Kopp and Robin Faillettaz collected the data. Alexa Abangan and Robin Faillettaz took the lead in the data analysis. Alexa Abangan, Dorothée Kopp and Robin Faillettaz wrote of the manuscript. All authors reviewed the analyses and edited the manuscript.

## Supporting information


**Data S1.** Supporting Information (SI 1–3). The experimental set‐up, technical specifications of the infrared lights, and the full‐length seabass trajectories.
**Figure S1.** The experimental set‐up of the infrared study with *D. labrax*.
**Figure S2.** The technical specifications of the infrared lamps used in the study (BERSUB® IR Wide).
**Figure S3.** Top view of the seabass trajectories during the entire infrared experiment per individual.
