# Supplementary material for: European seabass, Dicentrarchus labrax, show no significant response to infrared light
Source: J Fish Biol. 2025 Apr 1;107(2):652–7. doi: 10.1111/jfb.70038 (PMC12360150; doi:10.1111/jfb.70038)
Supplement: Supplementary file 1 — Data S1. Supporting Information (SI 1–3). The experimental set‐up, technical specifications of the infrared lights, and the full‐length seabass trajectories. Figure S1. The experimental set‐up of the infrared study with D. labrax. Figure S2. The technical specifications of the infrared lamps used in the study (BERSUB® IR Wide). Figure S3. Top view of the seabass trajectories during the entire infrared experiment per individual. [file JFB-107-652-s001.docx]

Supplementary Information (SI)


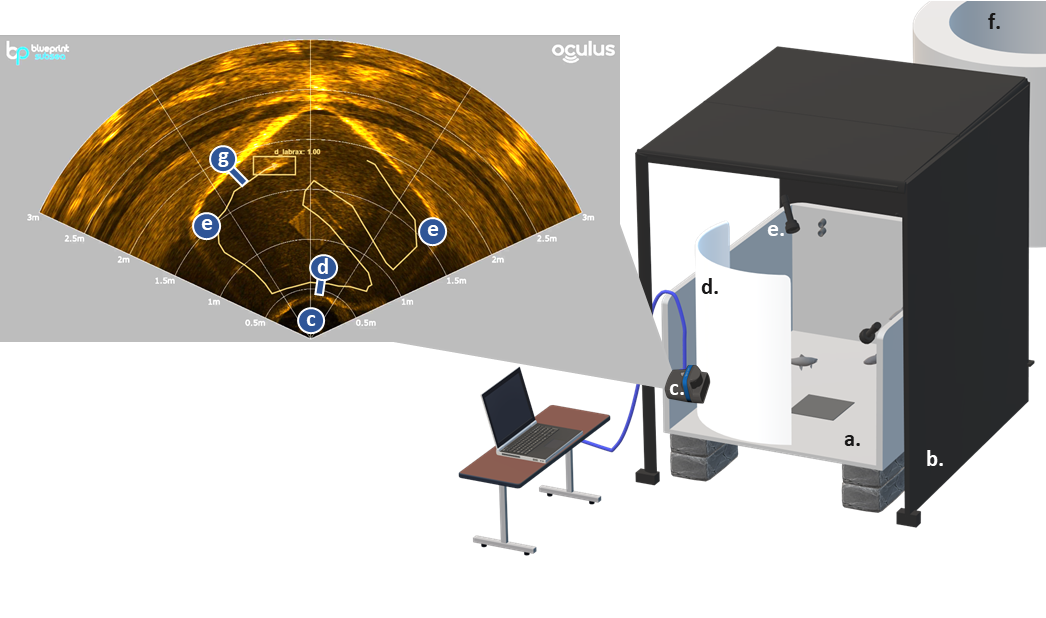


**SI Figure 1 (SI 1)**: The experimental set-up of the infrared study with *D. labrax*. **a)** the experimental basin, **b)** the basin cover that is impenetrable to any external light, **c)** the location of the hydroacoustic camera, **d)** the plastic barrier to protect the camera, **e)** the location of the infrared lights, **f)** the rearing basin where the individuals reside, **g)** a sample of a seabass trajectory.


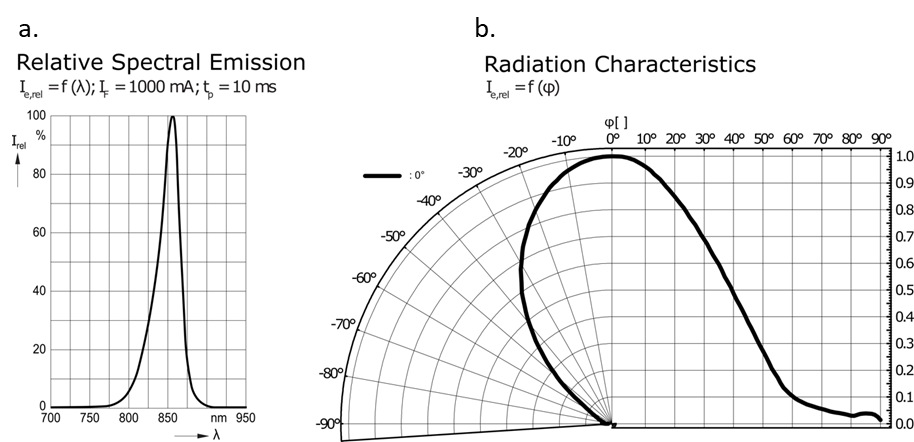


**SI Figure 2 (SI 2):** The technical specifications of the infrared lamps used in the study (BERSUB® IR Wide). **a)** The relative spectral emission showing the spectral range of the infrared light in nanometers. **b**) The radiation characteristics of the BERSUB IR lamp.


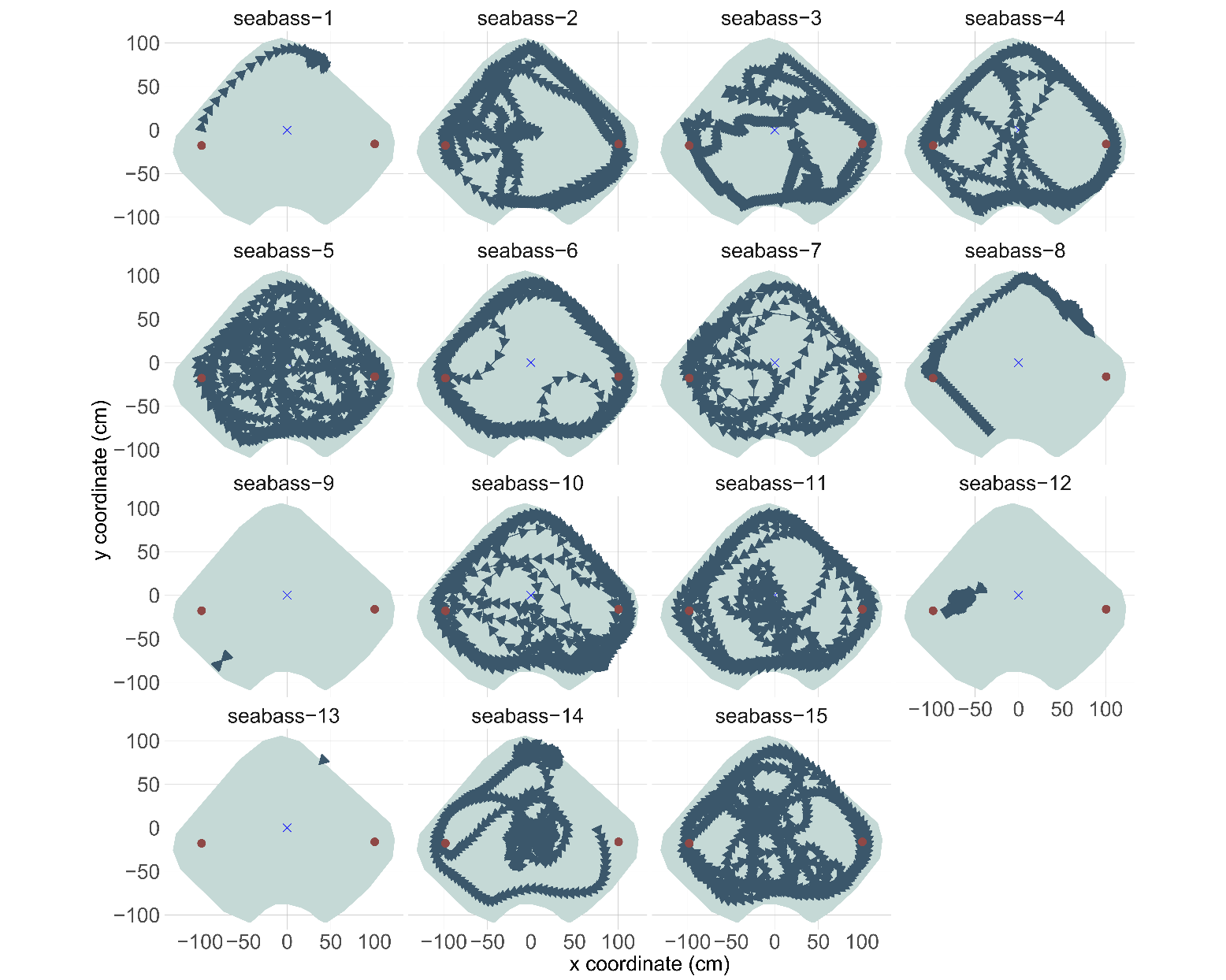


**SI Figure 3 (SI 3)**: Top view of the seabass trajectories during the entire infrared experiment per individual, where the **red dots** represent the infrared locations. The **blue cross** represents the center of the basin. The $x$ and $y$ axes represent the spatial coordinates in centimeters, with the center of the basin as origin ($x$=0, $y$=0). The nine individuals showing directionalities based on their positional angles are *seabass-2, seabass-3, seabass-4, seabass-5, seabass-7, seabass-10, seabass-11, seabass-14, seabass-15*. The five individuals displaying directionality based on their swimming direction are *seabass-2, seabass-3 seabass-7, seabass-14, seabass-15*.
